# DNA-GEL, Novel Nanomaterial for Biomedical Applications and Delivery of Bioactive Molecules

**DOI:** 10.3389/fphar.2020.01345

**Published:** 2020-09-04

**Authors:** Enrico Lattuada, Manuela Leo, Debora Caprara, Luisa Salvatori, Antonella Stoppacciaro, Francesco Sciortino, Patrizia Filetici

**Affiliations:** ^1^Department of Physics, Sapienza University of Rome, Rome, Italy; ^2^Institute of Molecular Biology and Pathology - CNR, Sapienza University of Rome, Rome, Italy; ^3^Department of Clinical and Molecular Medicine, Sant’Andrea Hospital, Sapienza University of Rome, Rome, Italy

**Keywords:** nanomaterials, DNA-GEL, gelation, biomedical applications, delivery of bioactive molecules

## Abstract

Novel DNA materials promise unpredictable perspectives for applications in cell biology. The realization of DNA-hydrogels built by a controlled association of DNA nanostars, whose binding can be tuned with minor changes in the nucleotide sequences, has been recently described. DNA hydrogels, with specific gelation properties that can be reassambled in desired culture media supplemented with drugs, RNA, DNA molecules and other bioactive compounds offer the opportunity to develop a novel nanomaterial for the delivery of single or multiple drugs in tumor tissues as an innovative and promising strategy. We provide here a comprehensive description of different, recently realized DNA-gels with the perspective of stimulating their biomedical application. Finally, we discuss the possibility to design sophisticated 3D tissue-like DNA-gels incorporating cell spheroids or single cells for the assembly of a novel kind of cellular matrix as a preclinical investigation for the implementation of tools for *in vivo* delivery of bioactive molecules.

## Introduction

**Figure 1:**
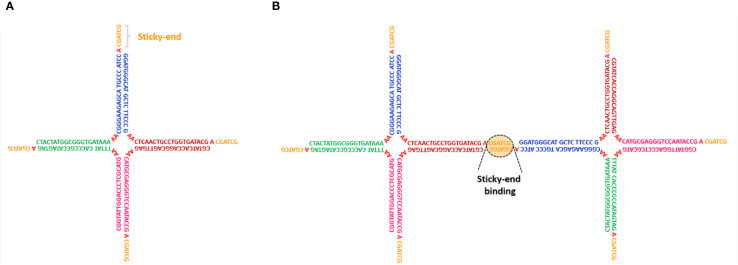
**(A)** Schematic representation of a limited-valence NS with functionality f=4, obtained by the self-assembly of four specific DNA sequences. Different colors highlight complementary DNA regions that selectively hybridize, forming the ds-sections that constitute the particle arm. Each arm terminates with a self-complementary sticky-end recognition sequence that allows NS-NS interactions **(B)**.

The well-understood reversibility and programmability of the Watson-Crick pairing interactions have promoted DNA as a leading component for the realization of bottom-up materials at the nanoscale (Seeman, 2016). The further support of robust synthesis methods, allowing to encipher a purposeful order of nucleotides in the DNA sequences, has encouraged the construction of several systems that respond to external stimuli such as temperature, pH, and presence of host DNA strands, among others (Murakami and Maeda, 2005; Condon, 2006; Cheng et al., 2009; Gao et al., 2015; Cherkasov et al., 2020). DNA has thus become an appealing material for the realization of 2D and 3D ordered structures (Winfree et al., 1998; Seeman, 2003; Zheng et al., 2009) as well as for the design of purposeful objects with predetermined shapes *via* the DNA Origami (Rothemund, 2006) or for the construction of hybrid materials that combine DNA with other systems, such as metallic nanoparticles (Macfarlane et al., 2011; Chao et al., 2015; Wang et al., 2015). When dissolving short DNA strands (oligomers) in an aqueous solution, segments of complementary sequences pair to form double-helical complexes, whose thermodynamic stability depends on the number of complementary bases. DNA nanotechnology exploits this general phenomenon by properly designing the oligomer sequences to build the desired structures. When the oligomers form an extended network, the resulting material is named DNA hydrogel. Such water-swollen DNA networks can reach macroscopic dimensions by mixing bulk quantities (≈100 μM) of oligomer sequences of nanometric size. The opportunity to play with a vast number of different oligomers makes it possible to generate DNA hydrogels differing in their local structure and functionality, from disordered networks resulting upon the spontaneous binding of randomly occurring sequences (Bellini et al., 2012) to the controlled formation of ordered networks composed of identical DNA nanoparticles, exploiting what is today called hierarchical multi-step self-assembly (**Figures 1A, B**). Typically, gelation stems from a two-step aggregation process. In the first step, the DNA oligomers pair to form suitably designed branched DNA structures. A possible example is shown in **Figure 1A**, where four selected sequences hybridize to form a nanostar with four arms. This aggregation process is followed by an additional step in which the binding of the sticky overhangs – single-stranded DNA sequences located at the end of each arm – occurs (**Figure 1B**). This mechanism allows for the realization of extended supramolecular structures with interesting behavior. The possibility to design specific bio-functional sequences with stimuli-responsive properties led to the construction of biocompatible DNA-hydrogels for biomedical applications, which are stable under physiological conditions. To date, these systems have been mainly applied to fields such as immunotherapy (Nishikawa et al., 2014; Ishii-Mizuno et al., 2017; Umeki et al., 2017; Shao et al., 2018; Komura et al., 2020), tissue engineering, drug and small molecules delivery (Um et al., 2006; Nishikawa et al., 2011; Nishikawa et al., 2014; Song et al., 2015b; Ishii-Mizuno et al., 2017; Umeki et al., 2017; Liu et al., 2018; Wei et al., 2019; Yue et al., 2019). Indeed, DNA-hydrogels represent a novel 3D scaffold in biomedical research, in addition to other natural and synthetic hydrogels - networks of polymers in an aqueous microenvironment - that have been developed and are very promising for biomedical applications (Tibbitt and Anseth, 2009; Gu and Mooney, 2016). These biomaterials, usually developed as injectable hydrogels, can be used depending on the specific biological purpose, from drug delivery (Ma et al., 2015; Pan et al., 2018) to tissue engineering for regenerative medicine (Keskar et al., 2009; Cheng et al., 2013; Purcell et al., 2014; Parmar et al., 2015). Therefore, structural, chemical, and mechanical similarities with the extracellular matrix (ECM) should be reproduced, such as the natural pore size, mechanical strength, cellular attachment, molecular response, biodegradability, biocompatibility, and solute transport (O’Brien, 2011; Thiele et al., 2014; Caliari and Burdick, 2016; Tsou et al., 2016; Naahidi et al., 2017). In the following, we will discuss, among others: the possibility to realize DNA-based materials that form a gel only around body temperature (Bomboi et al., 2016), which makes them ideal for the encapsulation of therapeutic oligonucleotides or drugs, as well as for favoring the interactions with specific proteins and RNA sequences; gels that do not cause an immune reaction, allowing the cell to live in the gel matrix for days (Um et al., 2006); gels with reconfigurable properties, able to restructure their topology *via* exchange reactions. Such systems are appealing for their self-healing and stress-realising properties, being thus promising for *in vivo* engineering applications, for cell transplantation and encapsulation (Um et al., 2006) as well as for the protein production (Park et al., 2009). The fight for cancer is coping with less toxic and more targeted therapy to avoid or lower the dangerous effects induced by the massive cytotoxic therapies. However, after aggressive therapies cancer cells often relapse with a worse and more aggressive malignant phenotype. In this context, Anakoinosis as an innovative therapeutic approach is intended to re-equilibrate tissue homeostasis. Current therapies, either classic antiblastic or more specific targeted therapies have the bias of selecting resistant tumor clones causing failure in the control of advanced disease. It is becoming increasingly evident that cellular damage induced by cancer progression is affected by tumor environment. Anakoinosis, the therapeutic approach based on reprogramming malignant cells in the frame of an intercellular communication and activation of endogenous signaling, has been proposed to achieve a more effective control of cancer cells progression. In this respect, there is an expanding research focused on finding the conditions and the effectors that can reactivate cellular homeostasis. In this field, DNA-gels might be applied successfully for their versatility. DNA-hydrogels will possibly be employed at preliclinical level in order to modify the intercellular communication and obtain a novel equilibrium in cellular homeostasis. DNA hydrogels composed of DNA NS can be dissolved in desired culture supplemented media, may incorporate different bioactive molecules, compounds, drugs and can provide an *in situ* platform for their targeted slow release inside tissues. In addition, the DNA-gel structure may also provide an ideal substrate for resetting the cellular homeostasis for redifferentiation of transformed cells. In this review, we discuss (i) the impact of DNA-hydrogels in cell environments, (ii) the potential applications of these materials, (iii) the *in vivo* analysis of DNA-gels and their applications to human cells, and (iv) possible future applications of these novel materials in the therapeutic field.

## DNA-Nanostars Hydrogels

**Figure 2:**
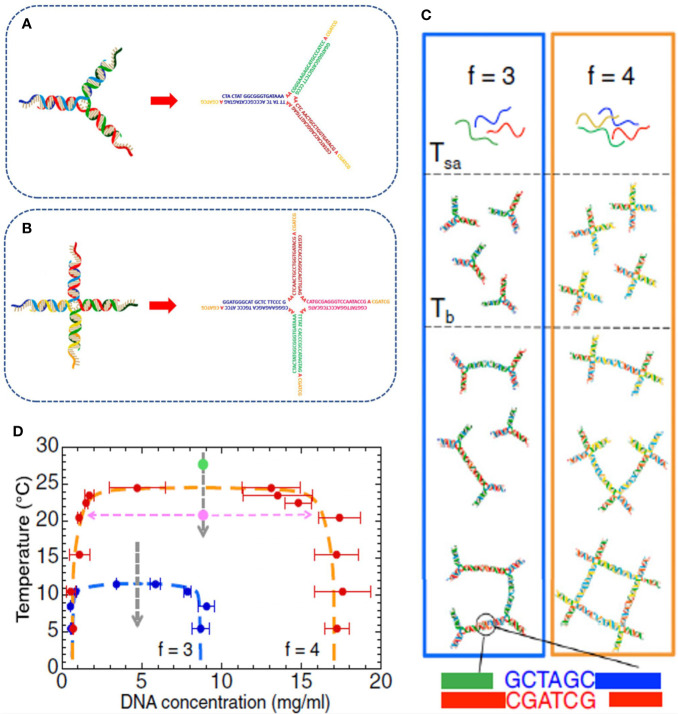
Experimental realization of limited-valence DNA particles of valence f=3 **(A)** and f=4 **(B)**, respectively, with the corresponding oligonucleotide composition. The particles are realized by self-assembling in solution three and four complementary DNA strands, respectively, that spontaneously hybridize into the desired arm-structures. **(C)** Temperature behavior of the two systems. At high temperature, the solutions are composed of freely diffusive DNA single strands; at intermediate temperature, the strands start to hybridize into the star-shaped geometries that, on further cooling, spontaneously assemble *via* the hybridization of the sticky sequences positioned at the end of each arm. **(D)** Phase diagram of the selected DNA NSs highlighting how the reduction of the valence shrinks the coexistence region to lower temperature and concentration values. Figure adapted from (Biffi et al., 2013).

In recent years, DNA self-assembly has been exploited to produce bulk quantities of nanometric particles of predefined shape and size. The possibility to take control at design time of the particle-particle interactions and built-in functionalities has led to several applications in the assembly of biodegradable and biocompatible DNA-gels with tailored properties (Um et al., 2006; Biffi et al., 2013; Bomboi et al., 2016; Nguyen and Saleh, 2017; Fernandez-Castanon et al., 2018). One important class of DNA constructs is represented by limited-valence DNA nanostars (NSs), that is, DNA nanoparticles with a finite number *f* of arms departing from a common central junction. These NSs have attracted increasing interest, encouraging the rational design of soft materials with unconventional behavior and tunable properties such as controllable shape (Um et al., 2006), kinetics, and structure (Nguyen and Saleh, 2017), permanent network structure formation *via* enzymatic reaction (Um et al., 2006; Xiang et al., 2016), re-entrant phase diagram (Bomboi et al., 2016), tunable viscosity (Bomboi et al., 2015), and self-healing (Bomboi et al., 2019). NSs can be experimentally produced by mixing in solution equimolar quantities of *f* purposely designed single-stranded (ss) DNA sequences, which are able to spontaneously self-assemble into a branched geometry with *f* double-helical arms. Particle flexibility can be increased by adding a small number of unpaired bases in the sequences at the location of the NS center. Finally, short single-stranded “sticky sequences”, placed at the end of each arm, promote particle-particle attractive interactions *via* strand hybridization. In the explanatory **Figure 1A**, we report an example of a tetravalent (*f*=4) NS with the particular DNA sequence design. The NS structure is composed of four double-stranded (ds) arms with a flexible core of 8 unpaired adenines, positioned at the center of the structure, which provide for particle flexibility. Each arm terminates with a self-complementary ss-sequence that allows NS-NS interactions (see **Figure 1B**). An additional unpaired adenine, positioned between the ds-regions and the sticky sequences, enhances the sticky-end flexibility facilitating the binding between different NSs. A series of studies focused on the phase behavior of DNA NS suspensions and their gelation properties, aiming at verifying how the functionality of the nanostars and the tunable external parameters (temperature, pH, salt-concentration, cosolute concentration) affect the probability of base paring and, consequently, how they can be exploited to control the (reversible) gel formation, its structure, and its viscoelastic properties. The emerging picture is consistent with recent predictions of the physics of patchy colloidal particles (Bianchi et al., 2011). Specifically, Biffi et al. (2013) studied the phase behavior upon temperature variation of trivalent (*f*=3) and tetravalent (*f*=4) DNA NS systems, formed by the DNA strands displayed in **Figure 2**. They showed that at high temperatures, around T ≈ 90°C, the system is composed of freely diffusing DNA single strands. Upon cooling, still at relatively high temperatures, the strands progressively assemble into star-shaped particles. The NSs do not show any relevant interaction, with the other NSs in solution, except for excluded volume and electrostatic repulsion. In an implicit solvent picture, the system behaves as a gas of weakly interacting particles. At sufficiently low temperatures, below the binding temperature of the sticky ends *T_b_* (the melting temperature of the sticky sequence), the NSs start to bind *via* the sticky tips on their arms, progressively assembling into larger aggregates up to the formation of a spanning network of bonded particles (gel state). The binding process, differently from a chemical reaction originating covalent bonds, is reversible so that the gel can be melted and reformed multiple times on changing the temperature. The authors also showed that a phase-separation (ps) phenomenon takes place in the system at low enough densities (when the concentration of DNA NSs is not sufficient to give rise to a connected network). When this is the case, particles separate into a NS-poor and NS-rich phase for temperatures below *T_ps_*. This process is very similar to the liquid-liquid phase-separation that forms membrane-less compartments in cells (Hyman et al., 2014) and could be fruitfully employed to create biocompatible chemical reaction zones (Nguyen and Saleh, 2017). Outside the phase-separation region, the gelation proceeds *via* equilibrium states in which the bond breaking and reforming allows stress release. Later, Bomboi et al. (2015) investigated the effect of the solution ionic strength on the phase behavior. In particular, they demonstrated that a higher NaCl salt concentration results in an increase of the temperature below which the network forms. Indeed, the larger concentration of ions screens the electrostatic repulsion of the NSs (due to the negative charge of the DNA phosphate backbone), facilitating the hybridization processes between complementary sequences. In line with experimental results, numerical simulations carried on by Rovigatti et al. (2014) showed that high salt concentrations broaden the phase-separation region to higher temperature and concentration values. The results of these studies can be summarized in the following points: (i) the binding temperature increases with the increasing length of the sticky-end sequence and the ionic strength of the solution. One must be careful to design the DNA sequences to guarantee a well-defined temperature-gap between the self-assembly of the NSs and the temperature at which the sticky sequences start to bind. By playing with the sequence, it becomes possible to generate systems that gel at the desired temperature (higher or lower than the body temperature, for example). (ii) The phase-separation concentration range shifts to higher values with the increase of the ionic strength of the solution, as the electrostatic repulsion between the NSs decreases. (iii) As the valence, *f*, of the NSs increases, both the temperature and concentration values of the phase-separation curve shift to larger values. This teaches us that gels with very low concentration of DNA can be created when the number of arms is small (*i.e.* functionality three). This behavior was also found in colloidal systems (for example, in patchy hard spheres), which constitute the simplest model of sticky NSs (Bianchi et al., 2006; Bianchi et al., 2011). Single component mixtures often provide sufficient control over the behavior of a DNA-gel. The addition of one or more components vastly increases their versatility and the range of applicability. By carefully selecting particles with different valence, one is able to assemble DNA-hydrogels with peculiar structural and dynamical properties.

### Re-Entrant DNA-Gel

**Figure 3:**
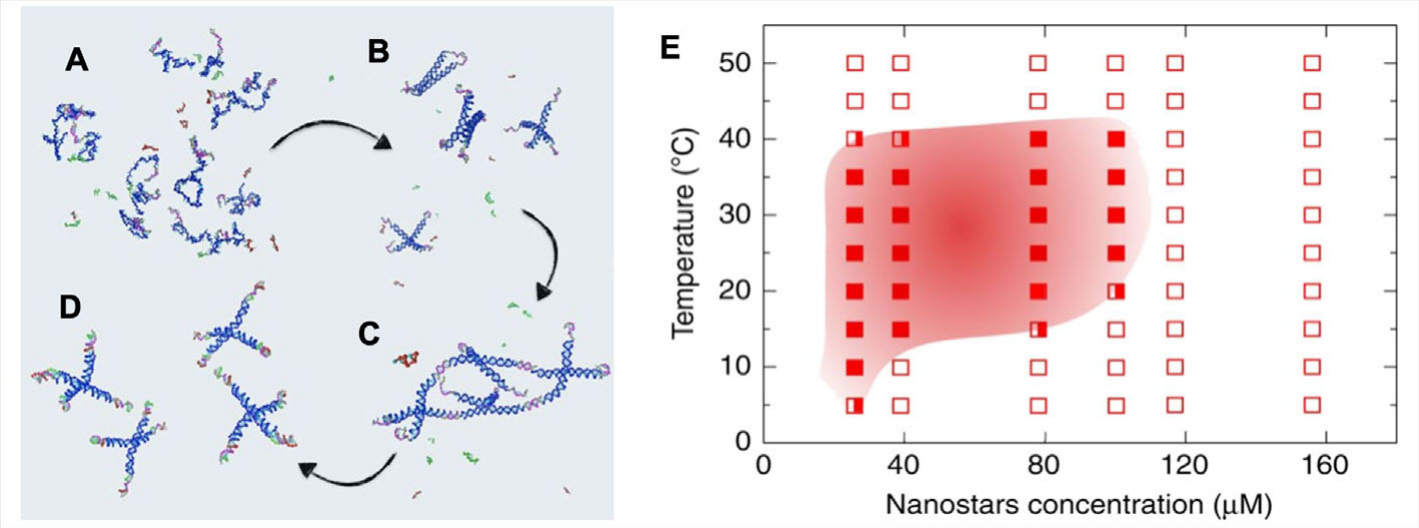
Re-entrant DNA gel. **(A)** At very high temperatures, the strands composing the A and B particles are all non-hybridized. **(B)** High-temperature behavior, the four strands comprising the A particles have now hybridized to form the tetravalent NSs, while the B particle sequences remain in their ss-configuration. **(C)** Intermediate-temperature behavior, different NSs bind *via* the sticky sequences to form a gel. The B particles have not yet hybridized. **(D)** Low-temperature behavior, where the B particles have displaced the AA bonds, originating free-floating AB_4_ clusters. **(E)** Phase-diagram of the re-entrant DNA system. Full squares denote phase-separated samples; open squares identify homogeneous solutions; half-full squares represent borderline cases. Figure adapted with permission under Creative Commons license CC BY 4.0 from (Bomboi et al., 2016).

Bomboi et al. (2016) used DNA NSs mixed with single DNA strands, which are able to cap the sticky overhangs below a specific temperature. They experimentally proved that one could obtain a suspension of tetravalent A-particles capable of assembling into a gel structure only within a specific range of temperatures, being fluid outside this selected temperature interval. The temperature range of this “circular” (re-entrant) phase diagram (see **Figure 3**) can be modified at design time by a careful selection of the DNA sequences. As the upper-temperature limit is given by the ability of the NSs to interact with each other, hence from the sequence encoded in the sticky-ends, the lower temperature below which the gel melts can be adjusted by the proper selection of the competitor ss-DNA sequence. Interestingly, the authors have demonstrated that this material can be fluid at room temperature and a viscous gel at body temperature.

### Swappable DNA-Gels

The physics of the bond swapping process (Zhang and Winfree, 2009) makes it possible to realize a reconfigurable all-DNA-gel system (Bomboi et al., 2019). The study provides proof that a smart design of the DNA sequences, able to self-assemble into tetra- and bi-valent nanoparticles, can generate a permanently bonded DNA-gel capable of swapping its bonds in a controlled way *via* toehold-mediated exchange reactions (Zhang and Winfree, 2009; Smallenburg et al., 2013; Srinivas et al., 2013). This peculiar behavior confers self-restructuring and stress-releasing properties to the gel, making it suitable for the implementation of tailored materials whose mechanical properties can be easily tuned by varying few external parameters (Montarnal et al., 2011). Besides, the non-toxicity and biocompatibility of the DNA nanoparticles make this system a promising platform for *in vivo* engineering applications.

### Stimuli-Responsive DNA-Gels

Temperature is not the only knob one can turn to tune the behavior of DNA-hydrogels. In fact, in biological applications, temperature control may not even be possible in principle since the environment is – to a certain extent – fixed to physiological conditions. The response to other external conditions can be tweaked, however, by smart modifications of the DNA sequences which account for the gel formation. Nucleic acids have been thus exploited as a flexible toolbox for the realization of many different intriguing systems.

#### Response to Environment pH

**Figure 4:**
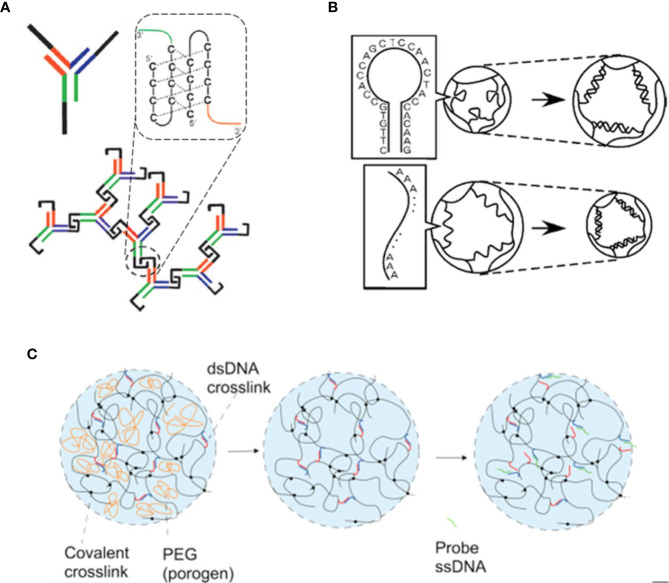
Examples of stimuli-responsive DNA-gels. **(A)** Schematics of a pH-responsive DNA system. Y-shaped DNA monomers bind to form a crosslinked hydrogel structure. The network formation is driven by varying the pH of the solution: at acidic pH values, the partial protonation of the C-rich domains (black) favor the hybridization between different monomers, as highlighted in the dashed box. Figure adapted from (Cheng et al., 2009). **(B)** Example of DNA hydrogels that can swell or shrink depending on the type of DNA crosslinkers. For stem-loop DNA crosslinks, the gel swells (top), while for linear ss-DNA intermolecular links, the gel shrinks (bottom). Figure from (Murakami and Maeda, 2005). **(C)** A DNA-polyacrylamide hybrid hydrogel with ds-DNA crosslinks able to differently respond to specific target ss-DNA sequences. The addition of polyethylene glycol (PEG) during the hydrogel preparation (left) - which is removed after the polymerization process (center) - allows the system to improve its swelling dynamics after the addition of a ss-DNA probe (right). Figure from (Gao et al., 2015).

DNA-hydrogels consisting of interconnected Y-shaped monomers (**Figure 4A**), able to pass from a fluid to a gel state in a reversible fashion by varying the pH of the solution, were engineered by Cheng et al. (2009). This peculiar behavior is achieved by properly designing the DNA monomers with ss-terminal interlocking domains, rich in cytosine. In a slightly acidic environment, the partial protonation of some cytosines favors the conjugation with the unprotonated cytosines belonging to a different domain, effectively linking two different monomers. At a proper monomer concentration, this leads to the formation of a spanning three-dimensional DNA-hydrogel. The further variation of the pH back to a basic solution allows restoring the free monomers configuration of the system.

#### Response to Species in Solution

In **Figure 4B**, we report a gel that can selectively respond to the presence of specific ss-DNA sequences in solution. Murakami and Maeda (Murakami and Maeda, 2005) showed that the network of a polymer gel could be made shrink or swell in the presence of ss-DNA sequences in the solvent if the crosslinkers (the bridges that keep the network glued) consist of purposely designed ss-DNA sequences. More precisely, by using as crosslinkers ss-DNA that hybridize into a stem-loop structure, the gel would swell when the target DNA in solution pairs with the sequence. In contrast, when DNA crosslinks maintain their linear geometry, the gel is found to shrink. Based on the same competitive displacement reactions, Gao et al. (2015) showed the experimental realization of a DNA-polyacrylamide hybrid hydrogel able to swell the network if a polyethylene glycol (PEG) agent is introduced during the preparation of the hydrogel and removed at the end of the synthesis. In such a case, the PEG acts as a pore-forming compound improving the swelling network dynamics that is activated by specific ss-DNA sequences (see **Figure 4C**). The proper design of hybrid hydrogels with selective responsiveness to sequences in solution could open many new pathways for biomedical applications and DNA-triggered sensing schemes. It might be also applied to load noncoding RNA molecules, such as microRNAs and long noncoding RNAs, active key regulators of cell differentiation and other cellular processes.

#### Photoresponsivity

An intriguing class of stimuli-responsive systems includes gels that selectively respond to light excitations. Such type of DNA-hydrogels can be realized by adequately modifying the DNA sequences with an azobenzene moiety, a light-responsive molecule that undergoes a *trans* to *cis* isomerization after irradiation with UV light and is able to return to the stable *trans* conformation when illuminated with visible light. The attractive possibility to convert such type of DNA-hydrogels into sol-gel states *via* reversible reactions, driven by the photo-regulated transition of the azobenzene, has been exploited by Kang et al. (2011) for the controllable encapsulation and release of several loads, including specific drugs, small molecules, nanoparticles, and proteins.

### DNA-Gels Joined by Ligation

**Figure 5:**
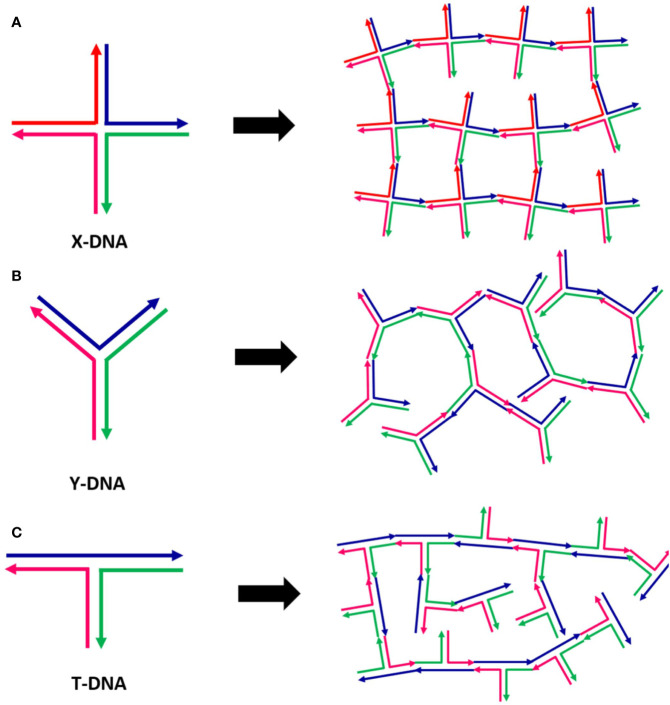
Examples of different DNA hydrogels realized by the assembly of specific DNA monomers of different shapes: X-DNA **(A)**, Y-DNA **(B)**, and Z-DNA **(C)**. Figure redrawn from (Um et al., 2006).

In 2006, Luo et al. experimentally realized anisotropic DNA nanoparticles with different shapes, able to self-assemble into different DNA-hydrogels (**Figure 5**) whose crosslinks have been made permanent *via* the use of the appropriate T4-ligase enzyme (Um et al., 2006). The tailored mechanical properties of the assembled network architectures can be changed at will by varying the type and concentration of the monomers. Besides, the exploitation of appropriate ligase-mediated reactions allows the gelation process to take place at physiological conditions. The intrinsic properties of DNA make these materials promising platforms for large applicability, in particular for *in vivo* encapsulation and release of specific drugs.

## Stability of DNA-Gels

In the clinical conditions, a ‘perfect’ nano-material should guarantee efficacy of the treatment and safety. In this respect, stability/degradation are important features to be considered when developing DNA-GEL for biomedical applications. Stability has been analyzed only *in vitro* with different results likely dependent on the type of DNA-gel. Um et al. designed a DNA-gel with tetra- and trimeric NSs bound by ligation (Um et al., 2006) which degradation *in vitro*, measured as the amount of DNA-gel/day in PBS at 37°C, was linked to the number of NSs branches. Over 70% of tetrameric NSs were still present after 14 days, while trimeric NSs were soon degraded in a few days. Interestingly, they found that the degradation was delayed if DNA-gel was loaded with drugs. DNA-hydrogel stability was also measured after DNase digestion (Nishikawa et al., 2014; Song et al., 2018). Although a variable degradation of DNA-hydrogel was reported (5-60%), higher stability of the DNA network with respect to free components was found. In other experiments, the stability was analyzed in serum to mimic cell culture conditions. However, the degradation of DNA-hydrogels was higher with high disassembly after 12hrs (Nishikawa et al., 2011; Wei et al., 2019).

## DNA-Hydrogels and Immune Response

The ability of the immune system to recognize external materials, with activation of the immune response by antigen-presenting cells and phagocytic cells (e.g. neutrophils, monocytes and macrophages) is well known (Chaplin, 2010). Nanomaterials were often found to induce the activation of the immune response at different levels, depending on properties like size, surface charge, among others (Adabi et al., 2017). This variability in response should be considered when designing new nanomaterials. The use of DNA-hydrogels is therefore of interest due to the low immunogenicity described in literature (Nishikawa et al., 2014; Umeki et al., 2017; Shao et al., 2018; Obuobi et al., 2019) and the possibility to functionalize them in order to boost the immune response in a controlled manner, with applications in vaccine and immunological therapy. Many DNA-gels have been designed with the insertion of CpG Oligodeoxynucleotides (ODN), synthetic oligonucleotides derived from bacteria, very effective in activating immune response (Klinman, 2004). Distinct classes of ODNs, composed of unmethylated CG dinucleotides flanked by a phosphorothioate backbone and palindromic regions, have been identified (Klinman, 2004; Scheiermann and Klinman, 2014) and tested, thus showing their high immunogenicity (Nishikawa et al., 2014; Ishii-Mizuno et al., 2017; Umeki et al., 2017; Shao et al., 2018; Komura et al., 2020). Shao et al. developed a vaccine, based on a self-assembled DNA-hydrogel of trimeric NSs and linker DNA containing CpG ODNs, functionalized with an antigen recognized by B and T-helper cells effective in the recruitment and activation of antigen-presenting cells (Shao et al., 2018). In murine macrophage-like cells, CpG DNA-hydrogels activation of cytokines was increased with respect to CpG ODN alone, thus suggesting that immobilization of CpG in the DNA network might improve their efficiency (Shao et al., 2018). Nishikawa et al. developed an injectable DNA-hydrogel (Nishikawa et al., 2014) functionalized with CpG ODNs and tested as a carrier of antigenic drug Ovalbumin (OVA), showing stimulation of immune response and increased activation of antigen-presenting cells in mouse macrophage-like cells. In mice, this DNA-hydrogel induced prolonged expression of cytokines at the injection site and not in serum, suggesting a local activation.

## DNA-Hydrogels for Delivery of Drugs and Nucleic Acids

Many efforts in nanomedicine have been made to develop carriers for drug delivery capable of overcoming the limits of free drug administration, such as low stability and insolubility in biological systems, short half-life, and systemic distribution (Yadav et al., 2020). As previously discussed, DNA-hydrogels are biodegradable and, in principle, the versatile nature of DNA sequences and their ability to respond to external stimuli allow their utilization for efficient encapsulation and release of drugs and other molecules. Several systems based on DNA-hydrogels made of NSs have been designed for loading and release of drugs (Um et al., 2006; Nishikawa et al., 2011; Nishikawa et al., 2014; Song et al., 2015b; Ishii-Mizuno et al., 2017; Umeki et al., 2017; Wei et al., 2019; Yue et al., 2019), nucleic acids, (Li et al., 2015; Nishida et al., 2016; Ma et al., 2018; Song et al., 2018; Komura et al., 2020), and proteins (Liu et al., 2018). The DNA-hydrogel can easily trap molecules by varying the stiffness and pore size of the DNA structure, as shown by Yue et al. (2019). The release of molecules can occur passively, through degradation of DNA (Um et al., 2006; Nishikawa et al., 2011; Nishikawa et al., 2014; Obuobi et al., 2019), or, actively, in response to external stimuli (e.g. when gold particles are heated upon irradiation with NIR wavelength) (Song et al., 2015a; Song et al., 2015b). The main achievement in nanomedicine is the development of nanocarriers for tissue and cell targeting reviewed in (Yu et al., 2016; Patra et al., 2018). In this case, the most used ligands are antibodies or peptides, though their selection and conjugation are difficult (Yu et al., 2016). An alternative is the use of DNA-hydrogels functionalized with aptamers (Li et al., 2015; Liu et al., 2018; Wei et al., 2019). In this perspective, DNA-hydrogels are suitable tools in the field of cancer therapy. The major limit in chemotherapy is the high toxicity of the treatment due to the systemic administration and the incapacity of chemo-drug to discriminate between normal and tumor cells (Ahlawat et al., 2018; Schirrmacher, 2019). Tumor environment shows increased permeability and acidity, and selected receptors are overexpressed on cancer cells allowing precise targeting (Ahlawat et al., 2018) and the above-mentioned characteristics of DNA-hydrogels well fit with cancer issues for the targeted delivery of chemo-drugs. Several DNA-hydrogels have been designed for release of Doxorubicin (DOX) (Nishikawa et al., 2011; Song et al., 2015a; Song et al., 2015b; Wei et al., 2019), which can be easily loaded into DNA-hydrogels for its interaction with DNA (Jawad et al., 2019; Mirzaei-Kalar et al., 2020). Among others, Wei et al. developed a sophisticated tumor-targeting DNA-hydrogel with a pH-controlled release of DOX (Wei et al., 2019). Recently, DNA-gels have been developed for the therapeutic release of nucleic acids (Li et al., 2015; Nishida et al., 2016; Song et al., 2018; Komura et al., 2020), such as small interfering RNA (siRNAs) for inhibition of target mRNA expression (Ahmadzada et al., 2018). siRNA systems have elicited interest in cancer therapy to suppress the expression of oncogenes. However, prototypes increasing the stability of siRNA must be developed (Kim et al., 2016). Song et al. designed a DNA-hydrogel producing siRNA (I-Gel) (Song et al., 2018) showing high silencing capability *in vitro* and a good cellular uptake without cell toxicity.

## Biological Effects of DNA-Hydrogels on Cell Cultures and Prospective Applications

The developing of DNA-hydrogels for biomedical applications requires the evaluation of their biological effects in human body tissues. The first step is the characterization of DNA-gels on cell cultures, which allows deep understanding of cellular and molecular mechanisms (Garcia et al., 2019). Moreover, the availability of cell lines derived from several tissues is useful to predict specific organ responses and develop tailoring protocols for different therapeutic applications (Adams and Brantner, 2006). However, while the studies on the physical structure and properties of DNA-gels are advanced, the application on cells is less investigated. Consequently, very little is known about the interaction between DNA-gels and cells and the possible cell specific response. One of the main problems in nanomedicine is cell toxicity caused by chemical and physical characteristics of nanomaterials (Sukhanova et al., 2018). To evaluate this issue and the interplay between DNA-gel and cells, preliminary characterization should consider the ability of DNA-hydrogels to affect basic cell properties like viability, proliferation and migration (Garcia et al., 2019). Currently, cell viability is the most analyzed parameter. It was found that DNA-gels did not cause alteration of viability in human dermal and tumor cell lines, and in mouse macrophage-like cells treated with trimeric or tetrameric NSs DNA-gels (Liu et al., 2018; Obuobi et al., 2019; Wei et al., 2019; Yue et al., 2019), and with NSs complexed with gold nanoparticles (Song et al., 2015a; Song et al., 2015b). The evaluation of cell death pathways is necessary, together with viability, since programmed cell death is a common outcome after nanoparticles exposure (Namvar et al., 2015; Sun et al., 2018; Fritsch-Decker et al., 2019). Wey et al. studied the effects of a DNA-gel composed of tetrameric NSs on cell viability and apoptosis in human breast cancer MCF-7 and lung adenocarcinoma A549 cell lines showing an increase in early apoptotic cells (Wei et al., 2019). Another question concerns the ability of NSs DNA-hydrogels to assembly in well-defined networks and the possibility that DNA-gels can be sensed by cells as physical stimuli, similar to artificial matrix used in cell culture applications (Trepat et al., 2012; Loh and Choong, 2013; Panzetta et al., 2019; Huang et al., 2020). In this view, the study of cell migration could be helpful to understand the interaction between DNA-gels and cells. Migration capability has been analyzed by Li et al. using a tetrameric NSs DNA-hydrogel containing an aptamer targeting lung adenocarcinoma A549 cell line (Li et al., 2015). They found that DNA-gel reduced cell migration on plates and a weak effect was shown even in the absence of the targeting aptamer. The above-mentioned works suggest the ability of DNA-hydrogel to influence basic cell properties. However, further studies are needed to better characterize the effect of DNA-gels on cell toxicity, cell cycle progression, proliferation and cell sensing in different cell models. Novel biological applications may take advantage from a choice of different DNA-hydrogel prototypes, however, their use in cell biology and tumors is at beginning due to their novelty. To this aim, the previously described re-entrant DNA-gel (Bomboi et al., 2016) is a very promising material for novel biomedical applications. Indeed, as above discussed, the re-entrant DNA-gel exhibits unusual phase-behavior, namely a fluid phase at 20°C and a gel state within a temperature range centered around the human body value (**Figure 3**). These peculiarities suggest that re-entrant DNA-gels might be used to encapsulate cells without detrimental effects. Actually, the possibility to encapsulate cells makes biomaterials promising tools for therapeutic applications, as they make it possible to retain cells in the site of injection and to replace or repair tissue damaged through trauma or disease (Cai et al., 2015). Mesenchymal (MSC), embryonic (ESC), and adult neural (NSC) stem cells have been widely used for this purpose. Their encapsulation into hydrogels was able to sustain cell growth and induce differentiation, with promising effects in tissue regeneration (Lei et al., 2011; Lim et al., 2012; Cheng et al., 2013; Kim et al., 2015; Xu et al., 2015). In addition, biomimetic scaffolds can also be used to reproduce sophisticated 3D culture systems to study cancer cells in more reliable conditions than conventional 2D models, through cell microenvironment engineering. Indeed, the features of the hydrogels, such as elasticity, permeability, adhesion, pore size, and biodegradability, can impact signal transduction pathways of cancer cells, particularly the growth and invasion ability as well as the response to therapies (Loessner et al., 2010; Ananthanarayanan et al., 2011; Liang et al., 2011; Szot et al., 2011; Beck et al., 2013; Del Bufalo et al., 2016). Therefore, *in vitro* engineered 3D tumors with defined microenvironment would provide novel information concerning the role of microenvironment in directing biological responses. Based on this information, the use of a re-entrant DNA-gel may be a novel opportunity to support medical treatment and therapies. In particular, among various possible applications, the interplay between the re-entrant DNA-gel and cancer stem cells, a small subpopulation of cells found in tumors and considered responsible for tumor onset and recurrence (Reya et al., 2001), could be an interesting field to explore. Cancer stem cells isolated from many types of tumor have the ability to grow as floating spheres *in vitro* (Salvatori et al., 2012; Favia et al., 2019) offering an additional model to investigate the effects mediated by re-entrant DNA-GEL. Moreover, as tumor spheroids better reflect the *in vivo* microenvironment than 2D cell cultures, they represent an appropriate model to evaluate signaling pathways and drug delivery (Mehta et al., 2012; Lee et al., 2013). Although Matrigel is often used as a scaffold for cancer cell spheres (Lang et al., 2001; Yeung et al., 2010; Badea et al., 2019), a re-entrant DNA-gel may be a new advance for spheroid growth and applications in cancer research, including cell response to therapies. Furthermore, cell-based therapy for regenerative medicine purposes could also benefit from the use of re-entrant DNA-gels. However, to be validated as a 3D scaffold for biomedical applications, it is mandatory for re-entrant DNA-gel to possess specific properties necessary to sustain the interaction with cells. The most important requirement is biocompatibility, which means that the material is able to support cell growth and that after implantation it does not induce an immune reaction (O’Brien, 2011). Hydrogels also need precise control over bioligand presentation in order to elicit desired cellular responses. Indeed, cell adhesion to ECM provides structural support and dynamic biochemical signalling regulating cell behaviors regarding the form, function, and fate of the cells, critical to tissue morphogenesis, homeostasis, and repair (Hynes, 2009; Rice et al., 2013; Schiller and Fassler, 2013). Biodegradability is another important factor to evaluate as the hydrogel should possess favorable degradation properties which provide the space for cell stretching, proliferation, and release (Kloxin et al., 2010; Lei et al., 2011; Schultz et al., 2015). Polymer concentration and crosslinking density are essential to determine hydrogel stiffness which can affect both stem cell pluripotency and differentiation, as well as cell migration (Leipzig and Shoichet, 2009; Musah et al., 2012; Kim et al., 2015). Adequate porosity of the scaffold is also needed for proper tissue regeneration. Indeed, although large pore size facilitates the transport of nutrients, too large pores may reduce cell infiltration due to the lack of contacts between cells and ECM. However, too small pores may trap cells, decreasing migration speed again (Dadsetan et al., 2008; Keskar et al., 2009; Teng et al., 2014). Following the many examples reported above, the main characteristics of the re-entrant DNA-gels will have to be investigated to validate this 3D scaffold as a novel tool in the integration of biomaterials engineering with the control of the aberrant homeostasis of cancer tissue, including cell reprogramming.

## Conclusion

Recent advances in DNA nanotechnology have allowed interdisciplinary cooperation among physical, biomedical, and chemical sciences, unlocking many new pathways for possible diagnostic and detection schemes. In particular, rationally designed DNA-hydrogels have garnered an increasing interest due to their versatility, cost-effectiveness, and biocompatibility, making them potential candidates for tissue engineering applications, self-healing, drug delivery, and cell culture. DNA-gels have been developed by physicists and material scientists. There is a lack of biological applications. DNA-gels might be successfully employed to obtain a targeted delivery inside tumor mass and preliminary ongoing applications on cells are extremely promising. DNA-gels show peculiar differences respect to classical substrates for cell cultures such as Matrix protein components. Among these, controlled gelation properties and the possibility to design sequences to obtain the desired structure with desired bulk behavior. In addition, DNA-gels can be supplied with nucleic acids such as microRNA, siRNA or DNA molecules. These peculiarities make DNA-gels an optimal matrix that might be used as a molecular selective filter to remove dangerous molecules such as oncomiRs or others expressed in the serum of cancer patients. Another application relies on the use of re-entrant gels that could be injected as a fluid at low temperature (20°C) and then become gelified inside the tissue at body temperature (37°C). Finally, the use of DNA-gels as a controlled environment where cancer cells can be included supplemented with desired effectors or molecules might be an additional possibility. In this respect it was interestingly shown that cell senescence, induced by low doses of chemotherapeutic drug, boost anticancer natural immune response in a Multiple Myeloma mouse model (Antonangeli et al., 2016), in this respect treatment of tumor cells with DNA-hydrogel might enhance the natural killer NK cells activity leading to eradication of cancer cells. We believe also that the 3D sponge-like structure of DNA-gels might represent a promising novel substrate for testing the cell behaviour in an intercellular microenvironment. Specifically, it is suitable to assay if selected drugs, hormones or other factors might be able to redifferentiate the cancer phenotype to obtain cells with a more physiological behaviour according to the Anakoinosis concept.

## Author Contributions

All authors contributed to the article and approved the submitted version. EL and ML contributed equally to writing and revision.

## Funding

The review has been supported by LAZIO INNOVA Regione Lazio, Progetto DNA-GEL Grant No. 85857-0051-0085.

## Conflict of Interest

The authors declare that the research was conducted in the absence of any commercial or financial relationships that could be construed as a potential conflict of interest.
